# Supplementary material for: Factors affecting guardians’ decision making on clinic-based purchase of children’s spectacles in Nigeria
Source: PLoS One. 2021 Jul 12;16(7):e0254517. doi: 10.1371/journal.pone.0254517 (PMC8274875; doi:10.1371/journal.pone.0254517)
Supplement: S1 File — (DOCX) [file pone.0254517.s001.docx]

| 1. **You decided to get your glasses here because:** | | | | | |
| --- | --- | --- | --- | --- | --- |
| **2. Did the staff at the clinic tell you about the different spectacle frames available? a) Yes b) No** | | | | | |
|  | **Excellent** | **Good** | **Neutral** | **Bad** | **Very bad** |
| **3 What do you think about the design of the spectacle frames available?** | **①** | **②** | **③** | **④** | **⑤** |
| **4 What do you think about the quality of the spectacles frames available?** | **①** | **②** | **③** | **④** | **⑤** |
| **5 When you were deciding which frame to purchase, which factor most influenced your choice?**  **a. Design ① Yes ②No If no, why? ________________________________________**  **b. Material ① Yes ②No If no, why? ________________________________________**  **c. Quality ① Yes ②No If no, why? ________________________________________**  **d. Price ① Yes ②No If no, why? ________________________________________**  **e. Recommendation from staff ① Yes ②No If no, why? ____________________________________**  **f. Brand choice ① Yes ②No If no, why? ________________________________________**  **g.Others, specify _______________________________________________________________________** | | | | | |
| **7 How could the current range of frames be improved?**  **a) Design b) Brand choice c) Material d) Others, specify _______________________** | | | | | |
| **8 For your child’s next pair of glasses will you return to the same eye clinic/hospital?**  **a) Yes, why?_______________________________ b) No, why?___________________________________** | | | | | |
| **9 Will you recommend you friends/relatives to the same eye clinic/hospital?**  **a) Yes, why?________________________________ b) No, why?___________________________________** | | | | | |
| **10 Why have you not get the frames from our clinic?** | | | | | |
| **11 Any further comments you might have?** | | | | | |

**Thank you very much for your time and participation. With your sincere response, we hope to improve our service and serve you better in the future.**
